# Surveillance of women at high risk for hereditary ovarian cancer is inefficient

**DOI:** 10.1038/sj.bjc.6603015

**Published:** 2006-02-21

**Authors:** A L Oei, L F Massuger, J Bulten, M J Ligtenberg, N Hoogerbrugge, J A de Hullu

**Affiliations:** 1 Department of Obstetrics and Gynaecology, Radboud University Nijmegen Medical Centre, PO Box 9101, 6500 HB Nijmegen, The Netherlands; 2 Department of Pathology, Radboud University Nijmegen Medical Centre, PO Box 9101, 6500 HB Nijmegen, The Netherlands; 3 Department of Human Genetics, Radboud University Nijmegen Medical Centre, PO Box 9101, 6500 HB Nijmegen, The Netherlands; 4 Department of Medical Oncology, Radboud University Nijmegen Medical Centre, PO Box 9101, 6500 HB Nijmegen, The Netherlands

**Keywords:** *BRCA*, surveillance, ovarian cancer, prophylactic surgery, hereditary cancer

## Abstract

To determine the effectiveness of annual gynaecological screening (pelvic examination, transvaginal ultrasound, and CA-125), a prospective cohort study of women at high risk for hereditary ovarian cancer was conducted. Women were offered DNA analysis followed by either annual screening or prophylactic bilateral salpingo-oophorectomy (BSO). Study population consisted of 512 high-risk women (median follow-up 2.07 years, range 0–9.4 years): 265 women (52%) had a *BRCA* mutation. Persisting abnormalities indicated diagnostic surgery in 24 women resulting in one primary ovarian cancer FIGO stage IIIc was found. The effectiveness of screening was studied by calculating the probability of finding ovarian cancers in the *BRCA-1* and *BRCA-2* carrier group and comparing this to the identified number of ovarian cancers. The number of ovarian cancer patients found at surveillance was in accordance with the predicted number of ovarian cancers. A total number of 169 women underwent prophylactic BSO: one ovarian cancer stage IIb was found. In conclusion, the surveillance programme for hereditary ovarian cancer does identify patients with ovarian cancer but is very inefficient considering the high number of surveillance visits and the advanced stage of ovarian cancer in the identified patient. For prevention of advanced stage ovarian cancer, prophylactic BSO from age 35–40 years is a more efficient alternative.

During the past two decades hereditary cancer has attracted a great deal of interest. Overall, the majority of cancers is sporadic, for example, only 5–10% of the ovarian cancers are hereditary (Garber and Offit, 2005). In general there is a suspicion of hereditary cancer when more than one primary tumour in the same patient is present, several first or second-degree relatives have cancer and/or cancer developed at an extremely early age.

In 1994 and 1995, mutations in the *BRCA-1* and *BRCA-2* gene were identified as a cause of hereditary breast and ovarian cancer (Scully *et al*, 1996; King *et al*, 2003). The lifetime risk of carriers for developing ovarian cancer is high, 35–60% for *BRCA-1* mutation carriers and 10–20% for *BRCA-2* mutation carriers, in contrast to the 1.8% lifetime risk of the general population (Ford *et al*, 1995; Antoniou *et al*, 2003). *BRCA* mutation carriers are also at risk for fallopian tube and primary peritoneal cancer, which usually presents with similar clinical features as ovarian cancer. Moreover, carriers have a 60–85% lifetime risk of developing breast cancer (Ford *et al*, 1995; Antoniou *et al*, 2003). Incidence rates for breast and ovarian cancer in *BRCA* mutation carriers have been estimated by Antoniou *et al* (2003).

*BRCA-1* and *BRCA-2* mutation carriers often participate in a surveillance programme for early detection of ovarian cancer consisting of annual pelvic examination and transvaginal ultrasound (TVU) combined with serum CA-125 assessment. As the prognosis of ovarian cancer is poor and the efficacy of this surveillance method has not been proven, prophylactic bilateral oophorectomy seems to be an attractive alternative. Since the discovery of an association between a *BRCA* mutation and the development of fallopian tube cancer, bilateral oophorectomy has been extended to bilateral salpingo-oophorectomy (BSO) (Zweemer *et al*, 2000; Olivier *et al*, 2004). Bilateral salpingo-oophorectomy (BSO) appeared to reduce not only the risk of ovarian cancer by up to 96%, but also the risk of breast cancer in pre-menopausal women by approximately 50% (Kauff *et al*, 2002; Rebbeck *et al*, 2002; Calderon-Margalit and Paltiel, 2004). Furthermore, Madalinska *et al* (2005) found no difference in quality-of-life between women who underwent prophylactic BSO and women who chose for periodic screening for ovarian cancer although women after prophylactic BSO had fewer concerns about developing breast and ovarian cancer. Whether or not to continue the surveillance after BSO, because of the remaining risk of primary peritoneal cancer, is subject to discussion.

The aim of this study is to evaluate the efficacy of surveillance for hereditary ovarian cancer at the Family Cancer Clinic at the tertiary referral centre of the Radboud University Nijmegen Medical Centre, The Netherlands.

## MATERIALS AND METHODS

### Study population

A total number of 512 women enrolled in the surveillance programme for hereditary ovarian cancer from January 1995 until January 2005 at the Family Cancer Clinic of the Radboud University Nijmegen Medical Centre, The Netherlands. Between 1995 and 1999 women enrolled in the surveillance programme through self-reference, reference by the department of human genetics, the general practitioner or other specialists. After 1999, women were first counselled at the department of human genetics and referred to the gynaecologist when indicated. Criteria for ovarian surveillance by the gynaecologist are described in Table 1. DNA testing was offered when a *BRCA* mutation was suspected based on criteria of the department of human genetics.

Primary surveillance was started at the age of 35 or 5 years earlier than the youngest family member with ovarian cancer. When a mutation was found in a hereditary breast and ovarian cancer family during the surveillance programme, all family members who did not carry the mutation were excluded from the surveillance program.

At primary surveillance, information on vital status, age of menarche, use of oral contraceptives, parity, breastfeeding, medical history, occurrence of cancer in the family and pre- or post-menopausal status, was collected. Furthermore, patients were counselled on the possibilities to manage their high-risk status. Their options consisted of annual surveillance or BSO.

### Annual surveillance

Surveillance visits were performed annually and included pelvic examination, transvaginal ultrasound (TVU) and serum CA-125 measurements. Transvaginal ultrasound (TVU) was abnormal in case of the following morphological abnormalities: multiple cysts, cyst with thick septa, papillary projection, irregular patterns or a variety in sonoluency. Cutoff value for CA-125 was 35 U ml^−1^. In case of an abnormal pelvic examination and/or TVU and/or CA-125, revision after 3 months was advised, unless the findings were highly suspicious for a malignancy in which case diagnostic surgery was performed. Initially, only the abnormal surveillance test was repeated. The other surveillance tests were repeated when the abnormal finding persisted and a diagnostic laparoscopy was considered. When diagnostic laparoscopy was unremarkable or only one ovary and/or tube was removed, the patient returned to the surveillance programme. Occasionally, TVU was performed without routine measurement of CA-125 and/or pelvic examination because the woman had recently visited her own gynaecologist at the referring hospital.

### Prophylactic bilateral salpingo-oophorectomy (BSO)

At primary counselling, patients were informed about the possible advantages (reduction of the risk of ovarian and breast cancer) and disadvantages (surgery, premature menopause, residual risk of peritoneal cancer) of a BSO and about the technical aspects (anaesthesia, risk of conversion into laparotomy). Patients who preferred BSO underwent laparoscopy and the ovaries as well as the fallopian tubes were removed. Hysterectomy was not part of the standard operation. All removed ovaries and tubes were separately histopathologically examined: a small piece of each ovary was frozen for further analysis in the future, macroscopic investigation for abnormalities and transverse sectioning of the ovary and fallopian tubes was performed. The tissue sections were formalin-fixed and paraffin-embedded. Standard 4 *μ*m thick haematoxylin and eosin-stained sections were used for histopathological examination.

After prophylactic BSO, women underwent follow-up for several years with annual CA-125 measurement. Since 2002 only one follow-up visit was performed and when no abnormalities were found the women were excluded from further control.

To check for the development of peritoneal cancer in any of the women who participated in our study the national pathology database (PALGA), in which all pathological diagnoses in the Netherlands are collected, was consulted for the existence of pathology reports with the diagnosis of ovarian, fallopian tube and peritoneal cancer of each woman until the first of January 2005.

### Statistical analysis

Data analysis was performed using SPSS 12.0.1 software packet (SPSS Inc. Chicago) and Microsoft Excel. The calculated incidence rates were based on incidence rates found by Antoniou *et al* (2003). Differences between characteristics were tested two-sided with the *χ*^2^-test with Yates’ correction for small numbers, when necessary. Differences in continuous characteristics, like age, were tested with the *t*-test. *P*-values⩽0.05 were considered significant.

## RESULTS

### Patient characteristics

The median age of all 512 women at enroliment in the surveillance programme was 42 years (range 20–75 years). An overview of the patient characteristics is given in Table 2. Ten women died during the surveillance period: seven died of breast cancer metastases, two died of massive lung embolism and in one case the cause of death was unknown. No patients died of ovarian cancer or primary peritoneal cancer.

### Ovarian cancer surveillance

A total of 1621 surveillance visits were performed. The median number of surveillance visits per patient was two (range 1–13 visits) and the median follow-up was 2.07 years (range 0–9.4 years) in a total follow-up of 1029 women-years. See Table 3 for the results of pelvic examination, TVU and CA-125 measurements. In the majority of initial abnormal diagnostic tests (343/378=90.7%), the abnormality could no longer be observed within 3–6 months.

Diagnostic laparoscopy due to abnormal findings at pelvic examination and/or TVU and/or CA-125 was indicated in 24 women. The median time between first surveillance and the diagnostic laparoscopy was 5 months (range 4–98 months). See Table 4 for an overview of the indications and outcome of these 24 diagnostic laparoscopies. Diagnostic laparoscopy was never indicated solely on a single abnormal pelvic examination (normal TVU and CA-125). The diagnostic laparoscopy procedure was converted to laparotomy in three cases (12.5%) owing to the size of the enlarged ovaries. One FIGO stage IIIc ovarian cancer was diagnosed during diagnostic surgery in a 52-year-old carrier of a *BRCA-1* mutation with a history of breast cancer.

During the surveillance programme 99 women out of the 364 women who underwent DNA analysis appeared not to carry a *BRCA* mutation. Thirteen of these women were members of a family in which a mutation was detected, 86 women were member of a family with no mutation but were at increased risk of ovarian cancer based on the family history. In retrospect, the 13 women without a mutation from a proven mutation family were not at high risk of ovarian cancer. These 99 women underwent 425 surveillance visits, seven had a prophylactic BSO and six had a diagnostic laparoscopy.

Incidence rates of the *BRCA-1* and *BRCA-2* mutation carriers based on incidence rates found by Antoniou *et al* (2003) are shown in Table 5. In our group of *BRCA-1* mutation carriers we calculated a 92% incidence rate in 248 woman-years. According to these calculations we predicted to find one ovarian cancer, which was subsequently observed. In 82 woman-years of the *BRCA-2* mutation carrier group a 8.7% incidence rate was calculated. In line with these calculations, we did not find an ovarian cancer case in this group.

### Prophylactic BSO

Prophylactic BSO was chosen by 169 women (169/512=33%). Of these 169 women, 149 (88.1%) were carrier of a genetic mutation: 104 *BRCA-1*, 44 *BRCA-2* and one woman was a carrier of both mutations. Twenty women without a *BRCA* mutation underwent a prophylactic BSO (seven had no mutation, the results of DNA analyses of five women were pending, seven women were not yet tested and in one woman the DNA status was unknown).

The median time between primary surveillance and BSO was 4 months (range 0–111 months). The median age of the women, who chose prophylactic BSO was 45 years (range 29–70 years). Eleven BSOs were converted to laparotomy due to intra-abdominal adhesions and/or otherwise not being able to resect the ovaries and tubes completely. One (0.6%) BSO was complicated by a wound infection. Of all BSOs, one (0.6%) FIGO stage IIb ovarian cancer was diagnosed in a 60-year-old *BRCA-2* mutation carrier with a history of breast cancer. The BSO was performed within one year after primary surveillance.

Final histopathological examination of the other ovaries and tubes showed one borderline malignancy, one dysplasia of the Fallopian tube and 31 cases with benign conditions, for example, serous cysts of the ovaries and endometriosis. All other histopathological examinations of the ovaries and Fallopian tubes were normal. Until the first of January 2005 no primary peritoneal cancers were diagnosed in these women after BSO.

## DISCUSSION

In this large, single-institution analysis, only two cases of ovarian cancer were diagnosed during annual surveillance for several years, among 512 women at high hereditary risk of ovarian cancer. One ovarian cancer (FIGO stage IIIc) was diagnosed at diagnostic laparoscopy after abnormal findings at the first surveillance visit in a 52-year-old patient with a history of breast cancer and carrier of a *BRCA-1* mutation. The other ovarian cancer (FIGO stage IIb) was diagnosed after BSO, which was intended to be prophylactic after normal surveillance 4 months earlier, in a 60-year-old patient with a history of breast cancer and carrier of a *BRCA-2* mutation. No interval cancers were identified.

The predicted number of ovarian cancers related to age and the presence of a *BRCA* mutation was calculated, using the incidence rates of Antoniou *et al* (2003). Based on these calculations the probability of finding one ovarian cancer in the *BRCA-1* mutation carrier group was 92% and the probability of finding one ovarian cancer in the *BRCA-2* mutation carrier group was 8.7% (see Tables 5 and 6). Judging on these calculations the probability of finding an ovarian cancer in the non-*BRCA* carriers is smaller than that for mutation carriers.

Based on the probability calculations and the findings during surveillance, we conclude that the surveillance programme does identify ovarian cancer patients, but is very inefficient for two reasons: (1) although the patient we identified at surveillance had advanced stage disease only the detection of early-stage disease will lead to an improvement of prognosis which is one of the primary goals of screening. (2) The detection of one patient with ovarian cancer requires disproportioned screening efforts in this group of high-risk women.

The cancer rates published by Antoniou *et al* (2003) are based on measurements obtained from a population that was tested for *BRCA* mutations regardless of family history. The women in our study were selected based on family history, which may have led to slightly higher incidence rates than the rates of Antoniou *et al* (2003).

Other ovarian cancer surveillance publications mainly report about patients with one affected family member (breast and/or ovarian cancer) (Bourne *et al*, 1991; Dorum *et al*, 1999). Limited data are available regarding women who enrolled in a study strictly on ‘high-risk’ criteria as shown in Table 1. This complicates comparisons between the different groups. See Table 6 for an overview of the most recent surveillance studies of high-risk women (TVU, CA-125 with (Dorum *et al*, 1999; Meeuwissen *et al*, 2005; Vasen *et al*, 2005) or without pelvic examination (Kauff *et al*, 2002; Laframboise *et al*, 2002; Scheuer *et al*, 2002; Stirling *et al*, 2005). Some studies report detection of advanced stage ovarian cancer, while a disappointing low number of patients appeared to have early stage cancer. Remarkably, only two of the studies cited in Table 6 reported interval cancers. Conversely, in other studies patients with a lower risk of ovarian cancer based on the family history were faced with interval ovarian cancers (van Nagell *et al*, 2000; Tailor *et al*, 2003). Stirling *et al* (2005) annually screened 1,110 high-risk women and only found two early stage (stage I) ovarian cancers. All other ovarian cancer patients had advanced stage disease. Meeuwissen *et al* (2005) calculated the sensitivity and specificity of this surveillance method and report a limited sensitivity with a high number of false-positive findings. Both groups also concluded that screening for ovarian cancer in high-risk women is highly ineffective.

A ‘wait-and-see’ protocol was followed regarding patients with abnormal findings during surveillance. This approach was supported by the high percentage (>90%) of abnormal findings, which normalized within 3–6 months. Pelvic examination did not have an additional role in detecting significant abnormalities in our patients. In all 1372 pelvic examinations, no isolated abnormality at pelvic examination indicated diagnostic laparoscopy. Apparently, pelvic examination does not have a diagnostic role and can be omitted during surveillance.

Most of the abnormalities at surveillance concerned TVU and/or CA-125 measurements. In 21.2% of all the TVU and 3.9% of all CA-125 measurements abnormalities were detected (see Table 3), probably due to the pre-menopausal status of the majority of women in our study. These women have a variety of both physiological (e.g. menstrual cycle) and benign conditions that can give rise to false-positive abnormalities on TVU (e.g. follicle cysts) or CA-125 measurements (e.g. endometriosis). This observation may have contributed to the high incidence of normalizing abnormalities.

In our study 69% of the carriers underwent BSO. This percentage is slightly higher than found in previous clinical studies, 58–60% (Kauff *et al*, 2002). The percentage of women choosing BSO increases with the age of women. Young women prefer to wait with prophylactic BSO until their family is complete. At the end of the study period, 88 (51%) of the 171 women who chose for screening were under the age of 40.

The observation that only one BSO patient had early-stage ovarian cancer combined with the low incidence of ovarian cancer in women after BSO confirms earlier reports about the effectiveness of prophylactic BSO in preventing advanced stage ovarian cancer. Rebbeck *et al* (2002) and Schwartz *et al* (2003) demonstrated that prophylactic bilateral oophorectomy reduces the risk of ovarian cancer with 85–95%. The observation of one case of ovarian cancer in 169 BSOs is lower than that was observed in other studies (Lu *et al*, 2000; Rebbeck *et al*, 2002; Scheuer *et al*, 2002). Until now BSO is the only evidence-based risk-reducing method for women at high risk of ovarian cancer. Moreover, BSO also reduces the risk of breast cancer in pre-menopausal women. Therefore, we would recommend counselling for prophylactic BSO from the age of 35–40 years in *BRCA* mutation carriers.

Limitations of our study include the non-randomized and observational design. However, randomizing is ethically impossible between surveillance and no surveillance. Moreover, the calculations using the incidence rates of Antoniou *et al* (2003) were very helpful in analyzing the effectiveness of surveillance. The data from our study are a representation of daily practice and give a true representation of this clinically relevant group. Studies with a larger group of *BRCA* mutation carriers and longer follow-up need to be performed before more definitive conclusions can be made.

In conclusion, we report one of the largest studies in women with a proven mutation at high risk of hereditary ovarian cancer in a single institutional setting. This surveillance programme identified one patient with advanced stage ovarian cancer. In retrospect, this reflects the predicted incidence rates for high-risk women in this age group. Apparently, this surveillance programme does identify patients with ovarian cancer but is very inefficient considering the high number of surveillance visits and the advanced stage of ovarian cancer in the identified patient. For prevention of ovarian cancer, prophylactic bilateral salpingo-oophorectomy from age 35–40 years is a more efficient alternative.

## Figures and Tables

**Table 1:** Criteria for referral to the gynaecologist for ovarian surveillance (Vasen *et al*, 1998; Sutcliffe *et al*, 2000)

**Criteria for referral**	
•	Women with a proven *BRCA-1* and/or a *BRCA-2* mutation.
•	Women from a family with a proven *BRCA* mutation but who are not (yet) tested for a mutation.
•	Women with first- or second-degree relatives with breast cancer before the age of 50, and ovarian cancer in the family.
•	Two first-degree relatives or one first-degree and one second-degree relative with ovarian cancer, independent of age.

**Table 2:** Patient characteristics of 512 women at primary surveillance

**Study population**		
Median age	42 years	Range 20–75 years
Median follow-up	2.07 years	Range 0–9.4 years
		
Type mutation no. (%)	Number	Percentage
*BRCA-1*	180	(34.2%)
*BRCA-2*	84	(16%)
*BRCA-1* and *BRCA-2*	1	(0.2%)
No mutation^a^	99	(19.1%)
Unknown	6	(1.5%)
Not tested	142	(27.3%)
		
Menopausal state	502	
Pre-menopausal	372	(72.7%)
Post-menopausal	130	(25.4%)
Missing	10	(1.9%)
		
Previous oral contraceptive use	199	(38.8%)
Previous pregnancy	403	(78.7%)
		
Previous breast cancer	165	(32.1%)
Previous abdominal surgery^b^	171	(33.4%)
Unknown	10	(1.9%)

a These patients underwent DNA testing during the surveillance programme and appeared to have no mutation.

b Abdominal surgery: for example, caesarean section(s), abdominal hysterectomy, salpingo-oophorectomy and appendectomy or other abdominal operations.

**Table 3:** Overview of initially abnormal findings at surveillance and follow-up

**Surveillance findings**	**Number of abnormal findings**	**Number of abnormalities that disappeared after 3**–**6 months**
Pelvic examination (*n*=1372)	11 (2%)	5 (45%)
CA-125 (U ml^−1^) (*n*=1278)	50 (4%)	43 (86%)
Transvaginal ultrasound (*n*=1493)	317 (21%)	295 (93%)
		
Total (*n*=4143)	378 (9 %)	**343 (91%)**

91% in bold emphasizes the percentage.

**Table 4:** Overview of indications and results of diagnostic laparoscopies in 24 women

**Abnormal finding(s) which led to diagnostic surgery**	**No of diagnostic laparoscopies**	**Outcome**
Abnormal pelvic examination	0	0
Abnormal CA-125 (⩾35 U ml^−1^)	1	1 – No abnormalities
Abnormal ultrasound	12	8 – Ovarian benign tumour^a^
		3 – No abnormalities
		1 – Haemorrhagic corpus luteum
Abnormal pelvic examination and CA-125 (⩾35 U ml^−1^)	1	1 – No abnormalities
Abnormal pelvic examination and ultrasound	4	4 – Ovarian benign tumour^b^
Abnormal CA-125 (⩾35 U ml^−1^) and ultrasound	5	1 – Salpingitis and paratubal cyst
		1 – Metastasis of breast cancer
		1 – No abnormalities
		**1** – **Ovarian cancer FIGO IIIc**
		1 – Endometriosis
Abnormal pelvic examination, CA-125 (⩾35 U ml^−1^) and ultrasound	1	1 – Metastasis of breast cancer

FIGO, International Federation of Gynecology and Obstetrics.

a In this group two patients had diagnostic laparoscopy converted to laparotomy.

b In this group one patient had diagnostic laparoscopy converted to laparotomy.

Ovarian cancer FIGO IIIc in bold emphasizes the finding of only one ovarian cancer.

**Table 5:** *BRCA-1* mutation carriers incidence during surveillance

	**Follow-up (years)**		**Group incidence (%)^a^**		**Total group incidence (%)**		**No surveillance visits**	
**Age group (years)**	***BRCA-1***	***BRCA-2***	***BRCA-1***	***BRCA-2***	***BRCA-1***	***BRCA-2***	***BRCA-1***	***BRCA-2***
20–24	4.37	0.76	0.001	0.001	0.004	0.001	9	6
25–29	38.54	2.65	0.002	0.002	0.077	0.005	69	10
30–34	80.05	30.16	0.18	0.004	14.409	0.120	115	43
35–39	75.61	20.06	0.28	0.01	21.170	0.200	158	34
40–44	25.51	6.45	0.87	0.08	22.193	0.516	53	18
45–49	12.15	12.79	1.49	0.14	18.103	1.790	26	29
50–54	5.12	4.38	0.96	0.6	4.915	2.628	19	12
55–59	3.54	3.60	1.19	0.75	4.212	2.700	13	12
60–64	0.80	1.53	2.26	0.38	1.808	0.581	3	5
65–69	2.09	0.30	2.49	0.42	5.204	0.126	4	1
>70	0.30			0.001			1	
								
Total	248.1	82.68			92.095	8.667	470	170

a Based on incidence rates of Antoniou *et al* (2003).

**Table 6:** Overview of literature: surveillance for ovarian cancer in high-risk women

**Study**	**Oei (2006)**	**Stirling *et al* (2005)**	**Meeuwissen *et al* (2005)**	**Vasen *et al* (2005)**	**Laframboise *et al* (2002)**	**Scheuer *et al* (2002)**	**Kauff *et al* (2002)**	**Dorum *et al* (1999)**
Mean age of patients in years (range)	40 (20–75)	Unknown	42 (18–77)	Unknown	47 (22–70)	47.7 (24.1–79)	45.5 (35–77.7)	41 (18–77)
								
No. of patients	512 high-risk women	1110 high-risk women	383 high-risk women	138 high-risk women	311 high-risk women	251 mutation carriers	170 mutation carriers	754 high-risk women
								
No. of patients with a mutation	180 *BRCA-1*	Unknown	127 *BRCA-1*	77 *BRCA-1*	26 *BRCA-1*	251 (distribution unknown)	104 *BRCA-1*	Not tested
	84 *BRCA-2*		25 *BRCA-2*	18 *BRCA-2*	5 *BRCA-2*		66 *BRCA-2*	
	1 *BRCA-1*							
	and *BRCA-2*							
								
No. of surveillance consults	1621	3701	1273	227	1555	Unknown (89 women)	350	Unknown
								
No. of diagnostic surgeries	24	29	20	36	9	10	11	Unknown
								
Incidence of cancer in women undergoing surveillance (FIGO stage)	1 ovarian	10 ovarian	1 × breast metastasis	6 ovarian	1 ovarian	4 ovarian	4 ovarian	16 ovarian
	1 × Stage IIIc	3 × Stage Ic		5 × stage III	Stage Ia	2 × Stage 1	1 peritoneal	06 × Stage I
		1 × Stage Iib		1 × stage IV		1 × Stage II		4 × Borderline stage I Others unknown
		1 × Stage Iic				1 × Unstaged		
		1 × Stage IIIb				1 peritoneal		
		3 × Stage IIIc						
		1 × Stage IV						
								
Incidence of interval cancers	0	2	2	0	0	0	0	0
								
No of patients choosing for prophylactic BSO	169	Unknown	133	97	Not performed	90	98	Not performed
								
Incidence of diagnosed cancers at prophylactic BSO	1 ovarian	1 ovarian	1 fallopian	1 ovarian	Not applicable	2 ovarian	1 peritoneal	Not applicable
	1 × Stage IIb	1 × Stage IIIa	tube	Stage Iic		2 × Stage I		
	2 breast metastasis		Stage Ia	2 fallopian				
			1 breast	tube				
			metastasis	1 × Stage I				
				1 × Stage II				

*Note*: All stages ordered in FIGO stage, International Federation of Gynecology and Obstetrics.
